# Supercritical CO_2_ Assisted Solvothermal Preparation of CoO/Graphene Nanocomposites for High Performance Lithium-Ion Batteries

**DOI:** 10.3390/nano11030694

**Published:** 2021-03-10

**Authors:** Ruoxin Yuan, Hao Wen, Li Zeng, Xi Li, Xingang Liu, Chuhong Zhang

**Affiliations:** State Key Laboratory of Polymer Materials Engineering, Polymer Research Institute of Sichuan University, Chengdu 610065, China; 2018323090018@stu.scu.edu.cn (R.Y.); wenhaowenh@163.com (H.W.); lizengzl@163.com (L.Z.); nicylixi@163.com (X.L.)

**Keywords:** lithium-ion battery, anode, supercritical carbon dioxide, cobalt monoxide, graphene

## Abstract

Supercritical CO_2_ (scCO_2_) is often used to prepare graphene/metal oxide nanocomposite anodes for high performance lithium-ion batteries (LIBs) by the assisted solvothermal method due to its low viscosity, high diffusion, zero surface tension and good surface wettability. However, the formation mechanism of metal oxides and the combination mechanism between metal oxides and graphene in this system are superficial. In this work, a cobalt monoxide/graphene (CoO/G) nanocomposite is fabricated via the scCO_2_ assisted solvothermal method followed by thermal treatment. We elucidate the mechanism that amorphous intermediates obtain by the scCO_2_ assisted solvothermal method, and then ultrafine CoO nanoparticles are crystallized during the heat treatment. In addition, scCO_2_ can promote CoO to be tightly fixed on the surface of graphene nanosheets by interfacial chemical bonds, which can effectively improve its cycle stability and rate performance. As expected, the CoO/G composites exhibit higher specific capacity (961 mAh g^−1^ at 100 mA g^−1^), excellent cyclic stability and rate capability (617 mAh g^−1^ after 500 cycles at 1000 mA g^−1^) when applied as an anode of LIB.

## 1. Introduction

With the increasing demand for recyclable energy, electrochemical energy storage devices that can effectively store the renewable energy sources of unstable output are in great demand, where lithium-ion batteries (LIBs) have been recognized as one of the most promising electrochemical energy storage devices [1,2]. However, the energy density of commercial LIB has met its bottle neck due to the low theoretical specific capacity of the commercial graphite anode (372 mAh g^−1^) [3], so it is urgent to develop anode materials with high specific capacity and energy density. Transition metal oxides acted as spotlights in the past decades due to their high theoretical specific capacity, vast choice of metal sources, and ease in controlling surface morphology [4,5,6,7,8]. Among them, Cobalt monoxide (CoO) has attracted increasing attention due to its high theoretical capacity (715 mAh g^−1^), highly reversible electrochemical reaction, low cost and high thermal stability [9]. However, the application of the CoO anode in LIBs is hampered by its poor cycling stability caused by large volume variation during the Li^+^ insertion/extraction, leading to electrode pulverization and electrical detachment from the current collector [10]. The mostly used paradigms to solve the above-mentioned problems are nano-sizing electrode particles combined with highly conductive buffer fillers [11,12]. Nevertheless, nano-sizing will lead to re-aggregation during cycling, which calls for tightly anchoring on the conductive substrate [13,14,15,16,17]. For the later strategy, hybridizing CoO with graphene not only facilitate electron transfer, but also inhibit the volume expansion of CoO during charge/discharge process due to the excellent electronic conductivity and superior mechanical robustness of graphene [18].

Up to now, many synthetic methods have been applied on preparing CoO/graphene composites, such as solvothermal [19], chemical reduction [20], solution phase coordination [17], and so on. Among them, solvothermal (or autoclave) method have been widely used because of its advantages such as high yield, simple manipulation, easy control, lower air pollution, and low energy consumption [21]. However, the inorganic particles obtained by the traditional solvothermal method are easy to agglomerate and are difficult to be uniformly distributed on graphene, resulting in unsatisfactory cycle stability. Supercritical CO_2_ (scCO_2_) has attracted wide attention due to its chemical inertness, non-toxicity, cost-effectiveness and environmental friendliness. The unique physical properties of scCO_2_, including low viscosity, high diffusion, zero surface tension, and good surface wettability make it recognizable as a promising alternative to traditional solvents [22,23,24,25,26]. Therefore, the scCO_2_ assisted solvothermal method has been widely used to prepare carbon-based composites for high performance LIB [27,28,29,30,31,32,33]. Due to the above unique physical properties, scCO_2_ can reduce the polarity of the solvent, so the nano-sized active materials can be evenly dispersed on the surface of carbon matrix, which greatly improves the cyclic stability of the nanocomposites. Although the role that scCO_2_ played in the formation of monodisperse nanoparticles has been discussed in literature [29,31,34], the formation mechanism of metal oxides and the combination mechanism between metal oxides and graphene in this system are vacant.

Herein, CoO/graphene nanocomposites (CoO/G-sc) were prepared by the scCO_2_ assisted solvothermal method followed by thermal treatment. Compared with the CoO/graphene composites prepared in air, the ultrafine CoO nanoparticles are uniformly dispersed on the surface of graphene nanosheets without agglomeration. In addition, XPS spectra show that there are more Co-O-C chemical bond in CoO/G-sc, indicating that the combination of CoO and graphene is more stable. Thus, the cycle stability, reversible capacity and rate performances of CoO/G-sc anodes are improved when applied as anodes in LIBs, which provides a reliable theoretical basis for the preparation of high-performance anode materials for a secondary energy storage system.

## 2. Materials and Methods

### 2.1. Reagents and Materials

Cobalt nitrate hydrate (Co (NO_3_)_2_·6H_2_O), graphite oxide powder, and absolute alcohol were purchased from Aladdin (Shanghai, China), Sixth Element Materials Technology Co., Ltd. (Changzhou, Jiangsu Province, China), and Chengdu Kelong Chemical Reagents Co., (Chengdu, Sichuan Province, China) respectively. All chemicals were used as received without further purification.

### 2.2. Preparation of CoO/Graphene Composites (CoO/G)

Graphene oxide (GO) colloidal was prepared by sonicating graphite oxide powder in ethanol for 2 h, and Co (NO_3_)_2_·6H_2_O) was added in above solution by 0.4 mol L^−1^. Then, as-prepared solution was transferred into a lab-assembled stainless steel autoclave of 50 mL. The autoclave was filled with CO_2_ up to 12 Mpa and maintained at 200 °C for 2 h. Then, the autoclave was slowly depressurized after it cooled down to room temperature naturally. The intermediate was filtered and then dried in vacuum at 80 °C for 6 h. For the sintering process, the obtained intermediate was calcined in a tube furnace under Ar at 450 °C for 2 h, which was denoted as CoO/G-sc. For comparison, samples also prepared without CO_2_, which was designated as CoO/G-a (a represents air).

### 2.3. Characterization

X-ray diffraction (XRD) patterns were recorded on a Rigaku (Smart lab III) using Cu Kα radiation within 2*θ* = 10–75°. Thermogravimetric analysis (TGA) was carried out on a TGA-Q50 (TA Instruments Co, Ltd., New Castle, DE, USA) at a heating rate of 10 °C min^−1^ from 30 °C to 800 °C under the air atmosphere. X-ray photoelectron spectroscopy (XPS) was conducted on an Escalab 250Xi X-ray photoelectron spectrometer (Thermo, Waltham, MA, USA). The morphology and microstructure were characterized from field emission scanning electron microscopy (FESEM, Quanta FEI, Hillsboro, OR, USA) and transmission electron microscopy (TEM, Tecnai FEI, Hillsboro, OR, USA). The chemical bond information was confirmed by Fourier-transform infrared spectrometer (FT-IR, Thermo, Waltham, MA, USA).

### 2.4. Electrochemical Measurements

The anodes were prepared by mixing CoO/G composites with acetylene black carbon and polyvinylidene fluoride (PVDF), at a weight ratio of 8:1:1, in the solvent of N-methyl-2-pyrrolidone (NMP). The obtained slurry was uniformly cast onto a copper foil and dried at 120 °C in a vacuum for 12 h. Then, the working electrodes were punched to diameter of 14 mm discs with about 1 mg active materials. Subsequently, CR 2032 coin-type cells were assembled in an argon-filled glove box. Lithium foil was used as the counter electrode, a porous polypropylene film (Celgard 2325, Charlotte, NC, USA) was used as separator and 1 mol L^−1^ LiPF_6_ dissolved in 1:1 (vol/vol) mixture of ethylene carbonate and dimethyl carbonate was used as the electrolyte. Galvanostatic charge-discharge measurements were done at various current densities in the voltage range of 0.1–3 V (vs. Li/Li^+^) using an automatic battery tester system (Land CT2001A, Wuhan, Hubei Province, China). Cyclic voltammetry (CV) tests were performed in the potential range of 0.1–3 V (vs. Li/Li^+^) with a scan rate of 0.2 mV s^−1^, and electrochemical impedance spectroscopy (EIS) measurements were carried out by applying an AC voltage of 5 mV in the frequency range of 100 kHz to 0.01 Hz using VMP3 electrochemical workstation (Biologic Science Instruments, Paris, France).

## 3. Results

**Figure 1:**
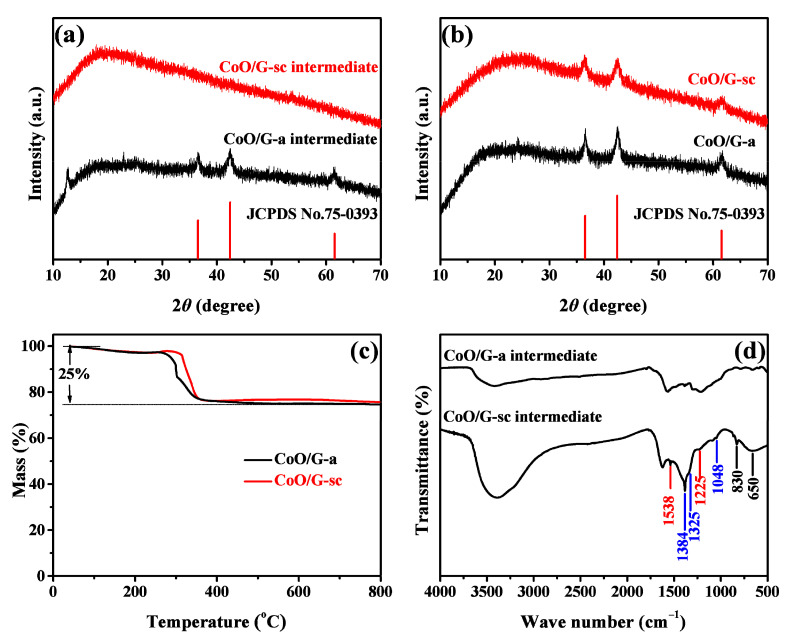
X-ray diffraction (XRD) patterns of (**a**) intermediates and (**b**) CoO/G-sc and CoO/G-a; (**c**) Thermogravimetric analysis (TGA) results of CoO/G-a and CoO/G-sc (**d**) FT-IR spectra of CoO/G-sc and CoO/G-a intermediates.

XRD measurements were employed to characterize the intermediate and final product prepared with and without scCO_2_. As illustrated in Figure 1a, intermediate synthesized without scCO_2_ shows a crystal phase of cubic CoO (JCPDS No. 75–0393). The other two peaks at 12.5° and 22.9° correspond to GO and graphene, respectively. While for the one prepared with scCO_2_, no crystalline diffraction peaks are found, suggesting an amorphous nature, which may be related with amorphous coordination complex containing CO_3_^2−^ and NO_3_^−^ [35]. After sintering, both samples are good agreement with the cubic phase CoO, and the characteristic peak of graphene (24.2°) can also be found, as depicted in Figure 1b. Average crystalline grain sizes are calculated as 8.5 and 11.5 nm for CoO/G-sc and CoO/G-a by applying Scherrer equation, suggesting that sample prepared with scCO_2_ has smaller grain size and crystallinity. Thermogravimetric analysis (TGA) was performed to evaluate fraction of CoO, as illustrated in Figure 1c. As shown, 25 wt% of the initial weight is lost in the temperature range 300–500 °C for both samples, which mainly derived from decomposition of graphene. Thus, the content of CoO can be calculated as 75 wt% for both samples. FT-IR patterns displayed in Figure 1d provide further information for understanding the forming mechanism of the two intermediates. Compared with CoO/G-a Intermediate, CoO/G-sc Intermediate shows stronger absorption between 500–1600 cm^−1^. The peaks at 1538 and 1225 cm^−1^ (red line) correspond to the asymmetrical vibration of ν_4_(−OCO_2_) and symmetric vibration of ν_1_(−OCO_2_) [36]; and peaks at 1384, 1325 and 1048 cm^−1^ (blue line) correspond to the asymmetrical vibration of ν_4_(-ONO_2_), asymmetrical vibration of ν_1_(−ONO_2_) and in-plane asymmetrical stretching vibration of ν_2_(−ONO_2_), respectively; the adsorption peaks marked as black lines centered at 830 and 650 cm^−1^ are related with out-plane bending vibration of ν_8_(−OCO_2_) and ν_6_(−ONO_2_) and in-plane symmetrical bending vibration of ν_6_(−OCO_2_) and ν_3_(−ONO_2_) [22,37,38,39,40]. In addition, the absorption peak of CoO/G-sc intermediate at 3950 cm^−1^ is stronger than that of CoO/G-a intermediate, which is attributed to the vibration of ν (H_2_O) [39]. It can be clearly found that there is a large amount of CO_3_^2−^ in the CoO/G-sc intermediate, but not in the CoO/G-a intermediate, which may be the reason that the CoO/G-sc intermediate is amorphous.

**Figure 2:**
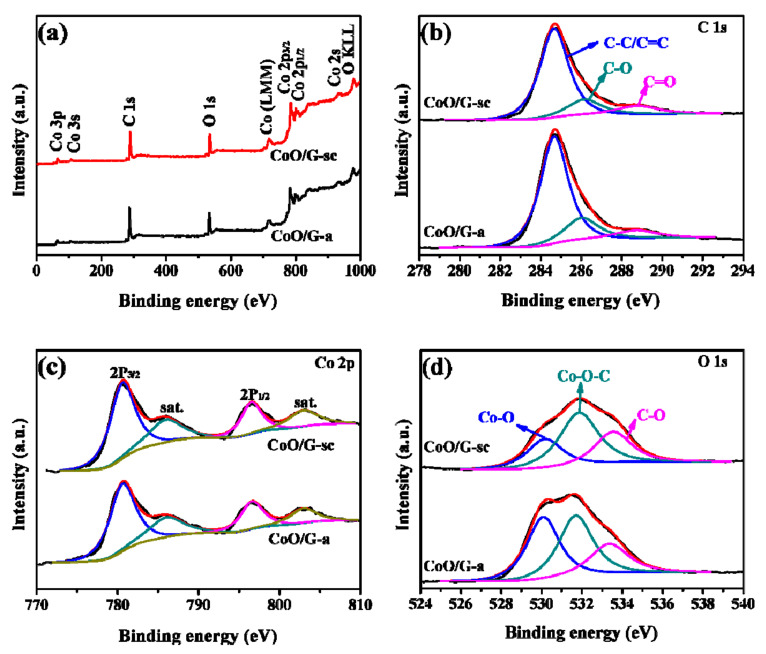
(**a**) X-ray photoelectron spectroscopy (XPS) spectra of CoO/G-sc and CoO/G-a; High-resolution (**b**) C 1s, (**c**) Co 2p and (**d**) O 1s XPS spectra of CoO/G-sc and CoO/G-a.

XPS was carried out to examine components and valence states of elements in synthesized samples. Similar patterns are displayed for CoO/G-a and CoO/G-sc in Figure 2a, with only the existence of C, O and Co. As shown in Figure 2b, the C 1s peaks of both samples can be fitted with three components: C-C/C=C (284.7 eV), C-O (286.2 eV) and C=O (288.8 eV), on which the latter two are related to the resident functional groups on graphene. The Co 2p_2/3_ (780.4 eV) and Co 2p_1/2_ (796.5 eV) peaks can be found in XPS spectra of Co 2p (Figure 2c), confirming the formation of CoO [17,41]. The O 1s peak also can be split into three components at 530.0, 531.7 and 533.4 eV, which related to the Co-O bond in CoO, residual oxygen on graphene and Co-O-C bonds between CoO and graphene, respectively. The existence of the Co-O-C covalent bond indicates a strong interaction between CoO and graphene [42,43,44]. The content of Co-O-C bonds in CoO/G-sc can be calculated as 48.6%, which is much higher than that of CoO/G-a (39.5%). This result strongly suggests that more CoO particles can be fastened tightly on the surface of graphene nanosheets with the help of scCO_2_. The massive Co-O-C bonds can facilitate fast transport of electron and Li^+^ between graphene and CoO particles, and stabilize CoO during charge/discharge cycles, ensuring that superior electrochemical performance of the CoO/G-sc electrode.

**Figure 3:**
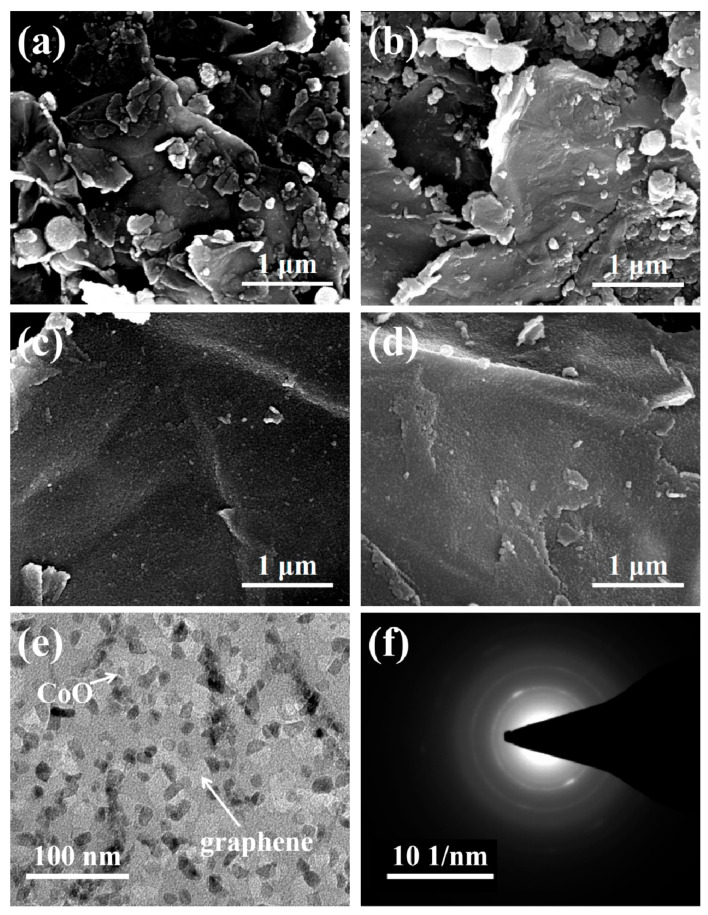
SEM images of (**a**) the intermediate and (**b**) final product of CoO/G-a; (**c**) the intermediate and (**d**) final product of CoO/G-sc; (**e**) Transmission electron microscopy (TEM) image and (**f**) Selected areal electron diffraction (SAED) pattern of CoO/G-sc.

**Figure 4:**
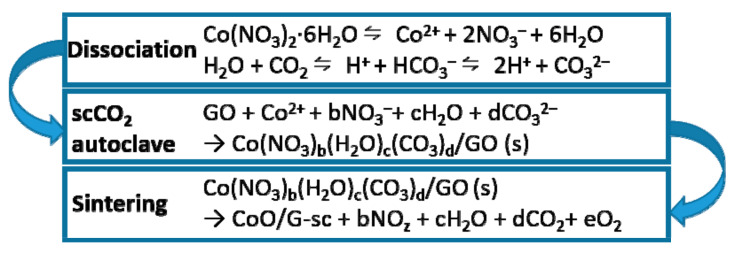
Schematic diagram of the formation mechanism of CoO/G-sc.

Figure 3a shows the surface morphology of the CoO/G-a intermediates, where the CoO particles agglomerate very seriously. After heat treatment, the agglomeration of CoO particles become more serious, and many large isolated CoO agglomerated particles are formed. For the CoO/G-sc intermediates, ultrafine particles are adhered uniformly on surface of graphene, as shown in Figure 3c. Even after heat treatment, there is no aggregation in CoO/G-sc sample (Figure 3d). The TEM image (Figure 3e) clearly shows that CoO nanoparticles with a size of about 10 nm are uniformly distributed on the graphene surface, and the SAED results indicate that CoO has good crystallinity. From the above results, we can conclude that scCO_2_ plays a crucial role in the formation and uniform distribution of monodisperse CoO nanoparticles during the solvothermal process. The probable reaction mechanisms [31,35] are proposed and illustrated in Figure 4. First of all, due to the unique physical properties of scCO_2_, the mixed solvent can completely infiltrate the graphene oxide nanosheets, thus ensuring that the Co^2+^ can be fully and uniformly dispersed around the nanosheets, which is the prerequisite for achieving uniform nucleation of the precursor on the graphene [23]. Secondly, CO_3_^2−^ generated by the reaction of scCO_2_ with water participates in the coordinated sedimentation and nucleation growth of Co^2+^, forming long-range disordered amorphous particles with low free energy on the surface of graphene, so can avoid the self-aggregation effectively [35]. Finally, after heat treatment, uniformly dispersed ultrafine CoO nanoparticles are formed on the surface of the graphene nanosheets.

**Figure 5:**
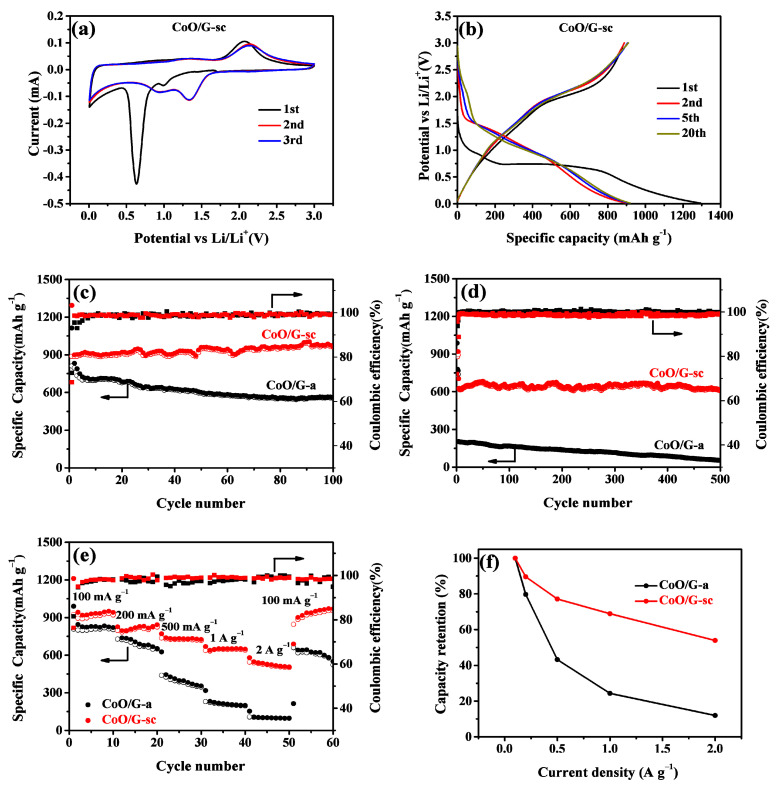
(**a**) cyclic voltammograms (CV) at a scan rate of 0.2 mV s^−1^ and (**b**) galvanostatic charge/discharge profiles of CoO/G-sc; Cycle stability and Coulombic efficiency of CoO/G-a and CoO/G-sc at specific current of (**c**) 100 mA g^−1^ and (**d**) 1 A g^−1^; (**e**) rate performance and (**f**) capacity retention ratio relative to 100 mA g^−1^ of CoO/G-a and CoO/G-sc electrodes.

CV and galvanostatic charge/discharge measurements were tested to investigate the electrochemical performance of the CoO/G-sc and CoO/G-a. Figure 5a shows the CV curves of CoO/G-sc with a scan rate of 0.2 mV s^−1^ between 0.1 and 3 V vs Li^+^/Li. In the first cathodic scan, two reduction peaks are observed centered at 1.0 V and 0.6 V, which are characters typical of CoO-based anodes. The peak at 1.0 V is attributed to the insertion of Li^+^ into CoO and the partial reduction of CoO (CoO + xLi^+^ + xe^−^→Li_x_CoO) and the latter one could be related to the formation of metallic Co (Li_x_CoO + (2x)Li^+^ + (2x)e^−^→Co + Li_2_O) and the formation of solid electrolyte interfaces (SEI) [45,46,47]. In the successive CV cycles, these reduction peaks move to a higher potential of 0.95 and 1.34 V because of smaller cathodic polarization [48]. In the first anodic scan, a broad peak located at 2.06 V appears, which can be attributed to the re-oxidation of Co to form CoO [46]. In addition, a pair of redox peaks could be found near 0 V, which could be the result of Li^+^ intercalation/extraction of graphene (C + xLi^+^ + xe^−^↔ Li_x_C). From the second scan cycle, the cathodic and anodic peaks reproduce very well, indicating that an excellent cyclic stability of the CoO/G-sc. For comparison, the CV curves of CoO/G-a (Figure S1a) show similar electrochemical reactions during the charge/discharge process but with inferior cyclic stability.

Galvanostatic charge/discharge curves of CoO/G-sc and CoO/G-a at a current density of 100 mA g^−1^ between 0.1–3 V vs Li^+^/Li are displayed in Figure 5c and Figure S1b, respectively. It can be observed that both electrodes show a discharge plateau around 1.0 and 0.7 V in the first discharge process, which is in accordance with CV results. In the following first charge process, a Co oxidation charge plateau located at 2.1 V is observed for both samples. The initial discharge and charge capacities of CoO/G-sc are 1294 and 887 mAh g^−1^, which are much higher than that of CoO/G-a (1113 and 810 mAh g^−1^). The higher specific capacity would be attributed to the nano-size of CoO particles, which brings more reaction site for Li^+^ storage. On the other hand, the nano-sizing brings a larger surface contact area between the electrolyte and the electrode, which leads to the formation of more SEI, resulting more irreversible capacity. Therefore, the initial coulombic efficiency of CoO/G-sc (68.6%) is lower than that of CoO/G-a (72.8%). Up to the 20th cycle, the specific discharge capacity remains about 919 mAh g^−1^ with a capacity retention of 71.0%, which is much higher than CoO/G-a (685 mAh g^−1^).

As depicted in Figure 5c, for the second cycle, the coulombic efficiency of CoO/G-sc is as high as 98.72%, and it remains stable in the following cycles. It is striking to note that the specific discharge capacity gradually increases in the following cycles, which is related to a reversible formation of a gel-like polymer membrane [45,49,50]. After 100 cycles, the reversible specific capacity reaches 961 mAh g^−1^, which is much higher than that of CoO/G-a (535 mAh g^−1^). For the CoO/G-a, the reversible specific capacities fading is associated with the repeat expansion and shrink of CoO aggregates. What is more, the large number of Co-O-C bonds in CoO/G-sc make CoO nanoparticles firmly anchored on the graphene surface, resulting in excellent cycle stability even at high current densities. Such cycle stability can also be demonstrated by SEM image after cycle, as negligible size change and agglomeration could be found (Figure S2) when compared with fresh sample. Therefore, the CoO/G-sc electrode can maintain a reversible specific capacity of 617 mAh g^−1^ after 500 cycles at a current density of 1 A g^−1^, which is 11.4 times that of the CoO/G-a electrode (Figure 5d). Moreover, the CoO/G-sc exhibits enhanced rate performance, as shown in Figure 5e. The specific capacity is maintained at values as high as 507 mAh g^−1^ even at 2 A g^−1^, and the capacity can be restored to 969 mAh g^−1^ when the current densities return to 100 mAh g^−1^, which indicates superior cycle stability and rate performances. The capacity retentions between high current density and initial 100 mA g^−1^ are calculated and illustrated in Figure 5f. Higher capacity retention is suggested for CoO/G-sc when compared with CoO/G-a in any current density. Even in a high current density of 2 A g^−1^, the value is 54% for CoO/G-sc, while the latter one is only 12%. We further compared the electrochemical performance of CoO/graphene composite electrodes reported in recent years. As shown in Table S1, the electrochemical behavior of our CoO/G-sc is significantly better than other CoO/graphene composites, which is mainly due to the uniform dispersion of ultrafine CoO and the large number of Co-O-C bonds with graphene.

**Figure 6:**
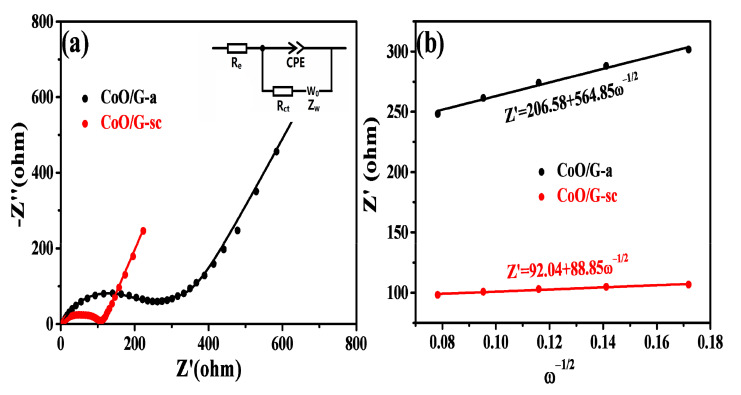
(**a**) Nyquist plots for CoO/G-a and CoO/G-sc after 5 cycles (inset: Equivalent circuit used to fit the experimental data), (**b**) Z’ vs. ω^−1/2^ plot in the low frequency region of CoO/G-a and CoO/G-sc electrodes.

The transport kinetics of the electrode were characterized by electrochemical impedance spectroscopy (EIS) measurements. Figure 6a and Figure S3 show the Nyquist plots of the two electrodes before and after 5 cycles, respectively. The semicircle for CoO/G-sc in the high/medium frequency range is much smaller than that of the CoO/G-a, indicating a lower contact and charge-transfer resistances for the CoO/G-sc electrode. The equivalent circuit is shown in the inset of Figure 6a, where Re is the ohmic resistance, Rct and CPE represent the charge–transfer resistance and the double layer capacitance, respectively, and Z_w_ is the Warburg impedance, which corresponds to the diffusion of the lithium ion into the bulk electrode. By applying equivalent circuits on the impedance data, the fitting results are displayed in Table 1. It can be observed that Rct and Z_w_ of CoO/G-sc are 96.1 Ω and 45.64 Ω s^−1/2^, which are clearly smaller than those of CoO/G-a (207.3 Ω and 462.8 Ω s^−1/2^), indicating better transmission for electrolyte and Li^+^, thus prompts good rate performances. The Li^+^ diffusion coefficient (D_Li_) could be calculated by applying Equation 1 [51,52], and the relationships between Z’ and ω^−1/2^ of two samples are shown in Figure 6b. Thus, the D_Li_ of CoO/G-sc can be calculated as 3.10 × 10^−13^ cm^2^ s^−1^, which is 2 orders of magnitude higher than CoO/G-a (7.68 × 10^−15^ cm^2^ s^−1^).

## 4. Conclusions

In summary, a solvothermal treatment with the assistance of scCO_2_ followed by a calcination process has been adopted to prepare a CoO/graphene nanocomposite. The formation mechanism of ultrafine nano-CoO was analyzed by FTIR, XPS, SEM, etc. The presence of scCO_2_ can control the deposition and nucleation of Co^2+^, so that CoO nanoparticles can be uniformly distributed and fixed on the graphene nanosheets by means of interfacial chemical bonds. When applied as an anode of LIB, the CoO/G-sc electrode exhibits a higher reversible capacity, excellent rate performance and cycling stability. The reaction route described herein offers a facile and green strategy for preparation of CoO/G composites as promising anode material for LIBs, which can also be applied to prepare many other graphene-based composite materials for energy storage applications.

## Figures and Tables

**Table 1:** Values of equivalent resistance used for fitting the experimental data.

Sample	R_e_ (Ω)	R_ct_ (Ω)	CPE (μF)	Z_w_ (Ω s^−1/2^)
CoO/G-a	5.79	207.3	9.5	462.8
CoO/G-sc	3.70	96.1	49.90	45.64

## Supplementary Materials

The following are available online at https://www.mdpi.com/2079-4991/11/3/694/s1, Figure S1: (a) Cyclic voltammograms at a scan rate of 0.2 mV s^−1^ and (b) galvanostatic charge/discharge profiles of CoO/G-a, Figure S2: SEM images of CoO/G-sc after 100 cycles at 100 mA g^−^^1^, Figure S3: Nyquist plots of CoO/G-a and CoO/G-sc before cycling, Table S1: Lithium storage performance comparison of CoO/graphene composite anodes from references.
