# Supplementary material for: Supercritical CO2 Assisted Solvothermal Preparation of CoO/Graphene Nanocomposites for High Performance Lithium-Ion Batteries
Source: Nanomaterials (Basel). 2021 Mar 10;11(3):694. doi: 10.3390/nano11030694 (PMC7999444; doi:10.3390/nano11030694)
Supplement: Supplementary file 1 [file nanomaterials-11-00694-s001.pdf]

Supercritical CO_2 assisted solvothermal preparation of CoO/graphene nanocomposites for high performance lithium-ion batteries

Ruoxin Yuan [1,†], Hao Wen [1,†]; Li Zeng [1], Xi Li [1], Xingang Liu [1,*]; Chuhong Zhang [1,*]
State Key Laboratory of Polymer Materials Engineering, Polymer Research Institute of Sichuan University, Chengdu 610065, China
Email: chuhong.zhang@scu.edu.cn
† These authors contributed equally to this work.

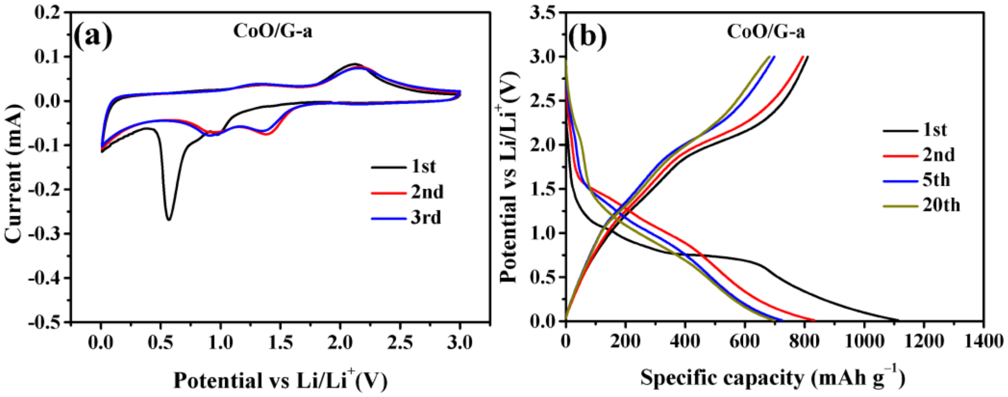

Figure S1 (**a**) Cyclic voltammograms at a scan rate of 0.2 mV s^{-1} and (**b**) galvanostatic charge/discharge profiles of CoO/G-a

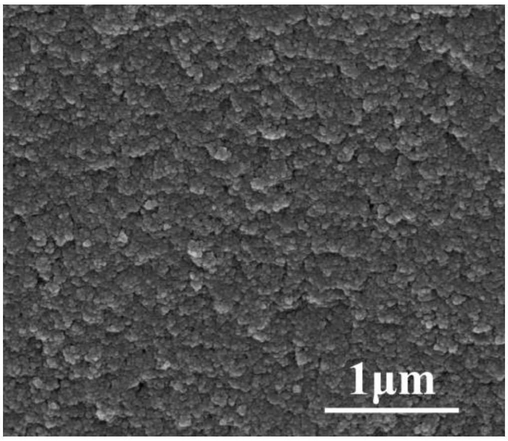

Figure S2 SEM image of CoO/G-sc after 100 cycles at 100 mA g^{-1}.

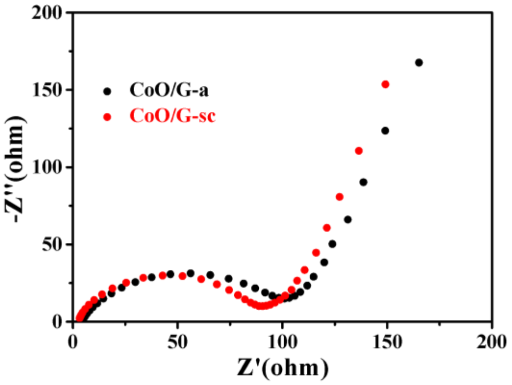

Figure S3 Nyquist plots of CoO/G-a and CoO/G-sc before cycling

Table.S1 Lithium storage performance comparison of CoO/graphene composite anodes from references

Sample	Cycling performance		Rate performance		References
	Current density (A g^{-1})	Cycle number/ specific discharge capacity (mAh g^{-1})	Current density (A g^{-1})	Specifc discharge capacity (mAh g^{-1})	
CoO/G-sc	0.1<br>1	100/961<br>500/617	2	507	This work
CoO-NPs/ graphene powder	0.05	50/650	0.5	340	[48]
CoO-HCs/ graphene powder	0.15	60/967	0.75	654	[49]
Ultrafine-CoO/ graphene powder	0.2	500/1018	1.6	531	[17]
CoO-NPs/ graphene powder	0.1	50/702	1.6	230	[50]
CoO-NRs/ graphene powder	0.1	50/960	1	513	[51]
CoO-NPs/ graphene aerogel	0.05	100/860	0.5	391	[52]
Ultrafine-CoO/ graphene powder	0.6	60/690	2.4	473	[20]
CoO-NPs/ graphene film	0.1	150/640	3	~450	[53]
CoO-NPs/N-doped graphene aerogel	1	500/450	1.6	373	[19]

NPs: nanoparticles, HCs: hollow cubes, NRs: nanorods.

~ means no detail value was given by references, and was evaluated via figures.

The Li^+ diffusion coefficient (D_{Li}) could be calculated by applying Equation 1:

$$D_{Li^+} = \frac{R^2 \cdot T^2}{2A^2 \cdot n^4 \cdot F^4 \cdot C^2 \cdot \sigma^2} \qquad \textbf{1}$$

Where R represents ideal gas constant (8.314J mol^{-1} K^{-1}), T represents temperature when testing (298K), A is the area of electrode (0.95cm^2), n is for the electron transfer number while electrochemical reaction occurs (2mol), F is for Faradic constant (96,500 C mol^{-1}), C is the concentration of Li^+ (10^{-3}mol cm^{-3}), and σ is Warburg coefficient, which is related with import sinus wave frequency.

$$Z' = R_D + R_L + \sigma\omega^{-1/2} \qquad \textbf{2}$$

$$\omega = 2\pi f \qquad \textbf{3}$$

Where RD and RL are fixed for each electrode, ω could be obtained by linear regression of Z′ (real part of resistance Z) vs $\omega^{-1/2}$, and the value of slope is σ. The Warburg coefficient of two electrode were calculated
